# Supplementary figures and images for: Damped Cantilever Microprobes for High-Speed Contact Metrology with 3D Surface Topography
Source: Sensors (Basel). 2023 Feb 10;23(4):2003. doi: 10.3390/s23042003 (PMC9967852; doi:10.3390/s23042003)

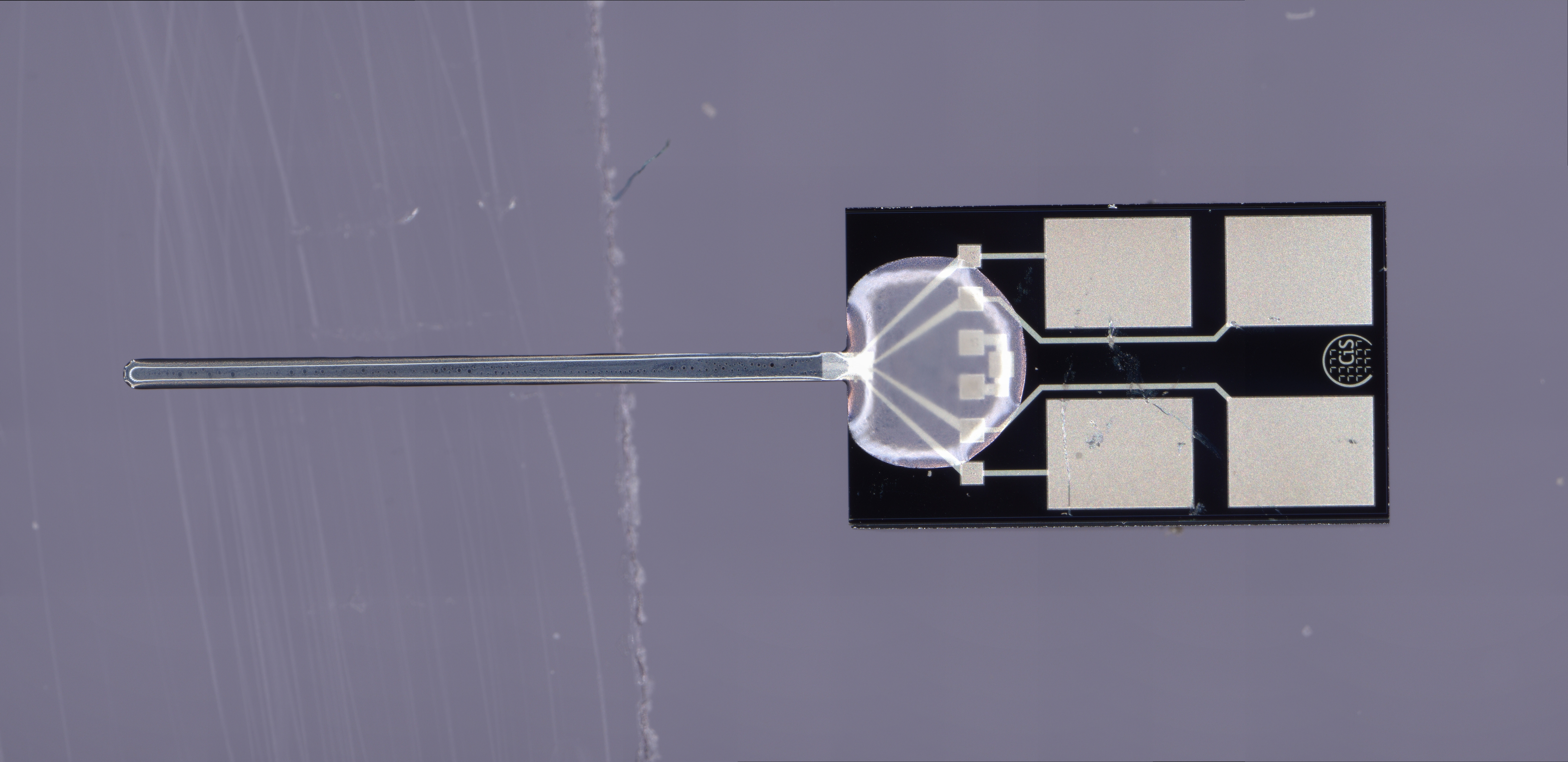

Supplement: Supplementary file 1 [file sensors-23-02003-s001.zip › Figure S1.jpg]

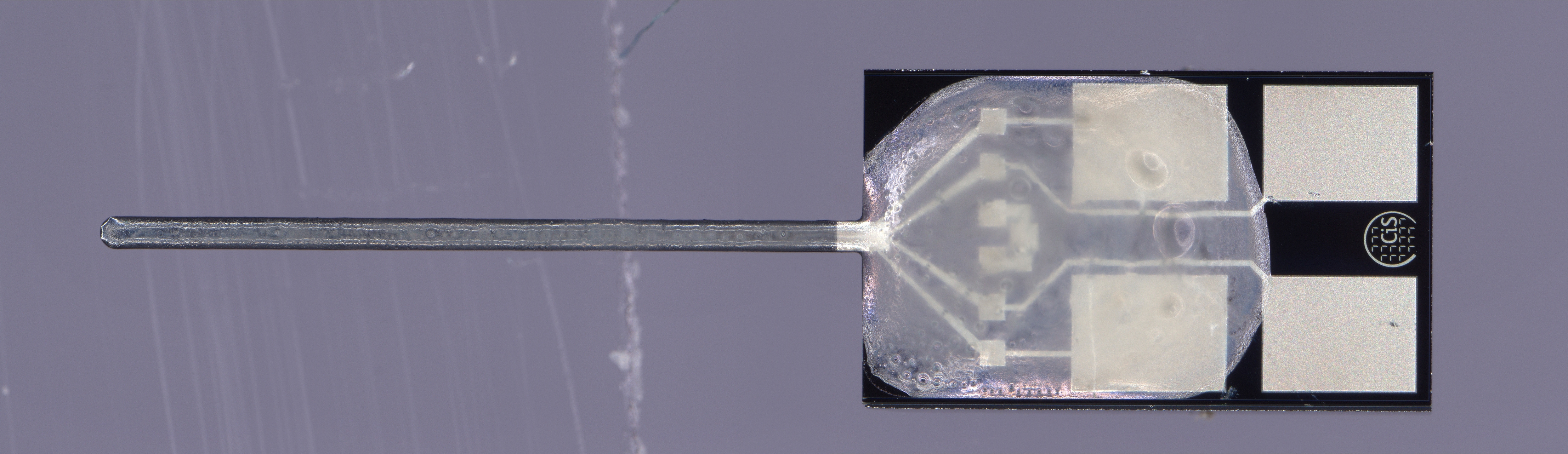

Supplement: Supplementary file 1 [file sensors-23-02003-s001.zip › Figure S10.jpg]

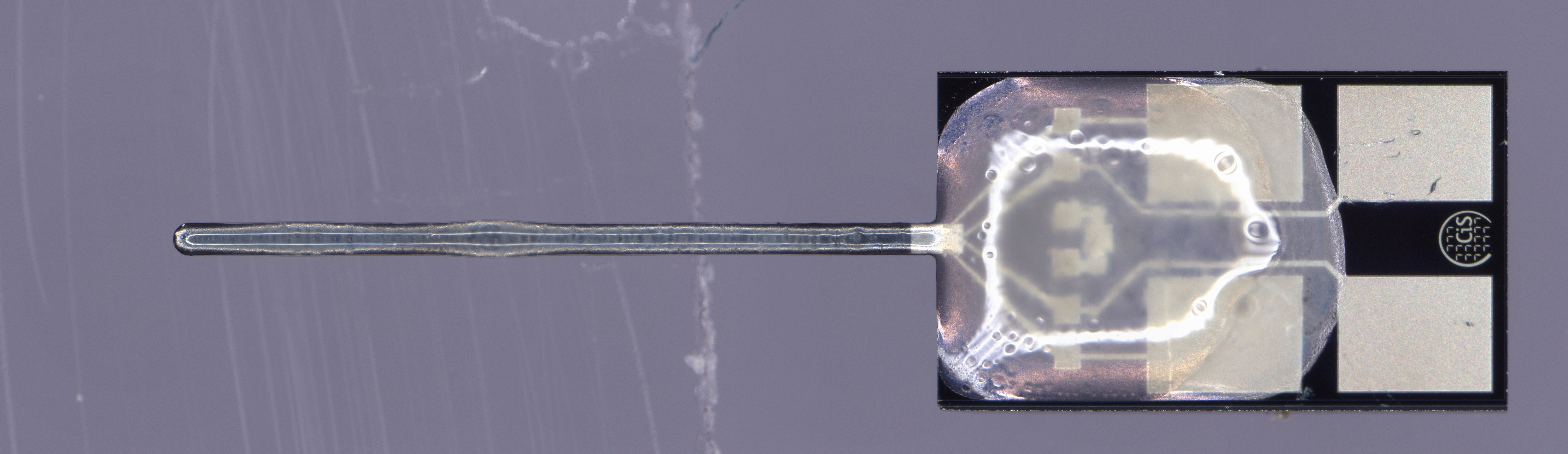

Supplement: Supplementary file 1 [file sensors-23-02003-s001.zip › Figure S11.jpg]

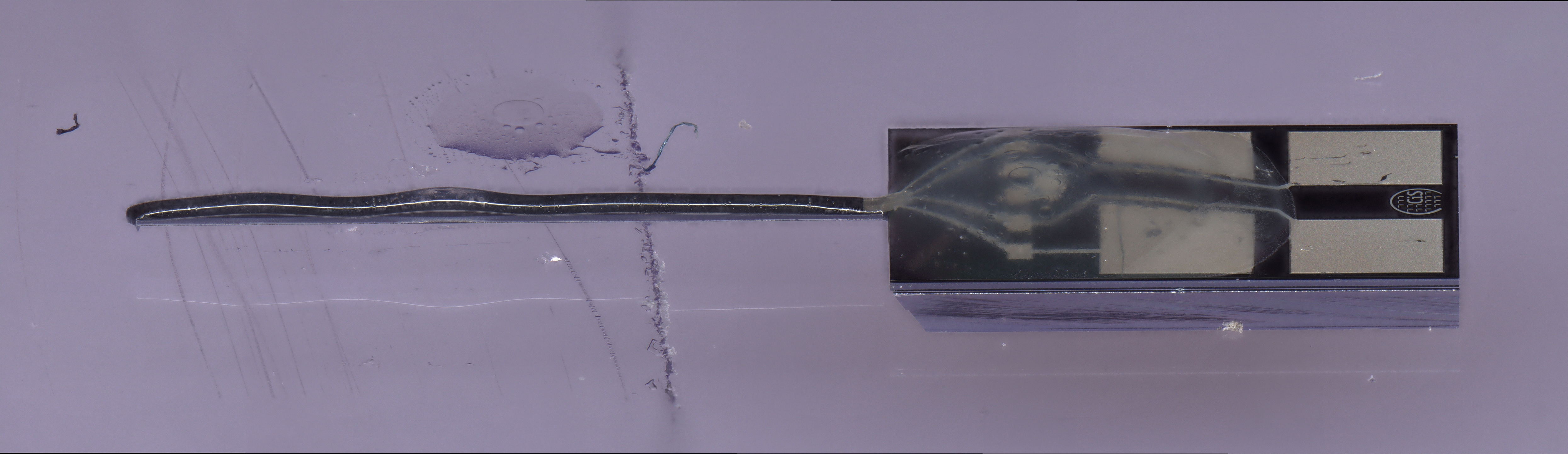

Supplement: Supplementary file 1 [file sensors-23-02003-s001.zip › Figure S12.jpg]

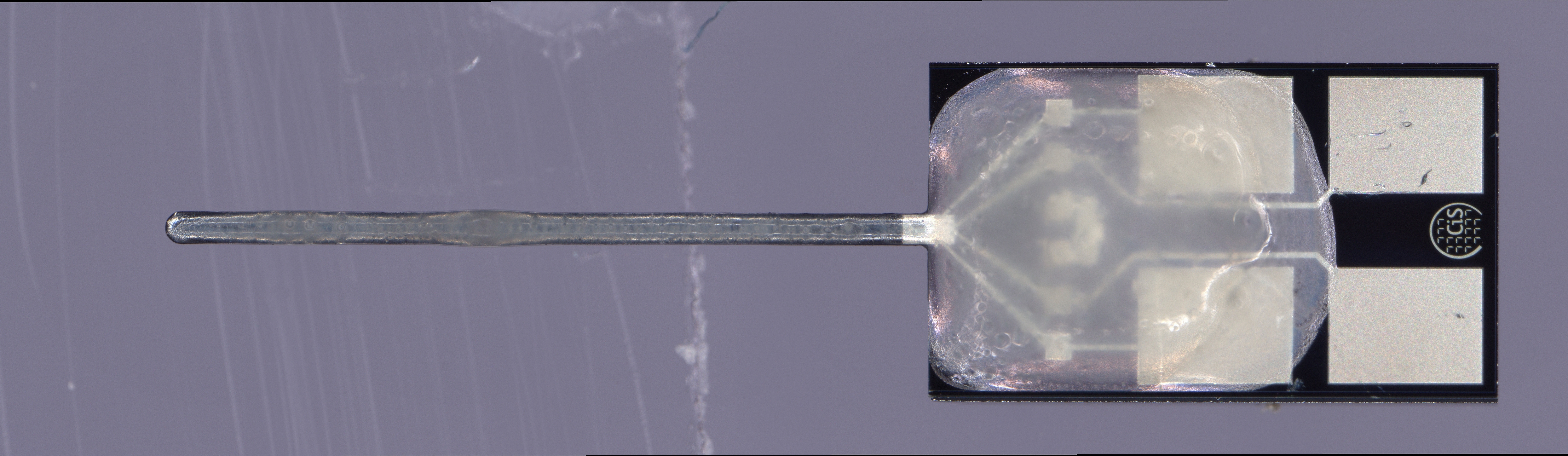

Supplement: Supplementary file 1 [file sensors-23-02003-s001.zip › Figure S13.jpg]

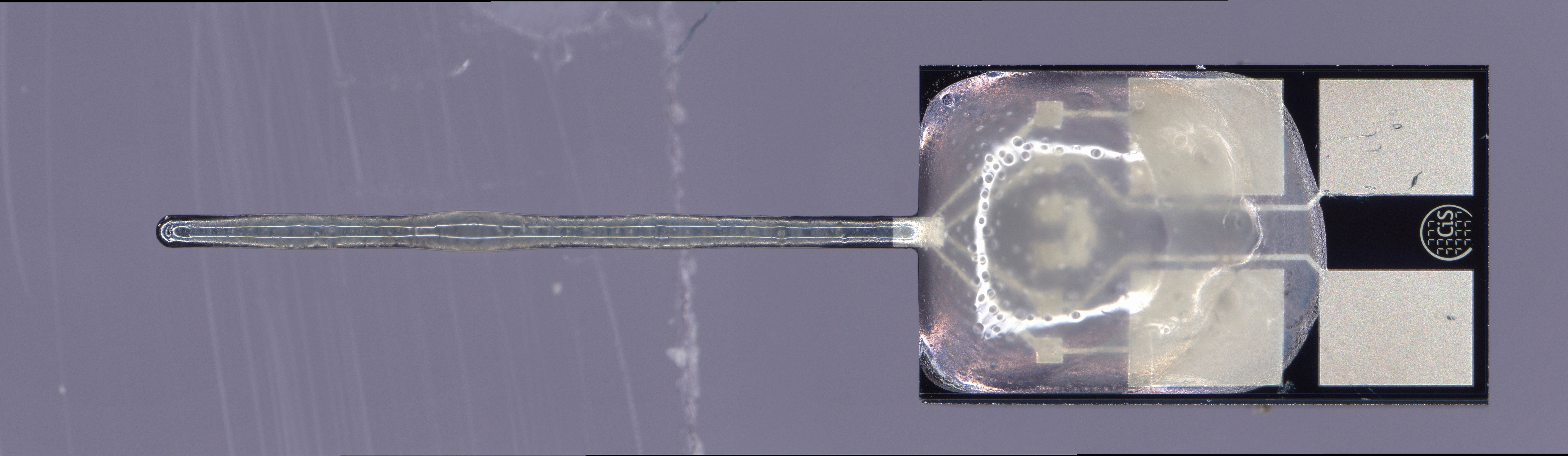

Supplement: Supplementary file 1 [file sensors-23-02003-s001.zip › Figure S14.jpg]

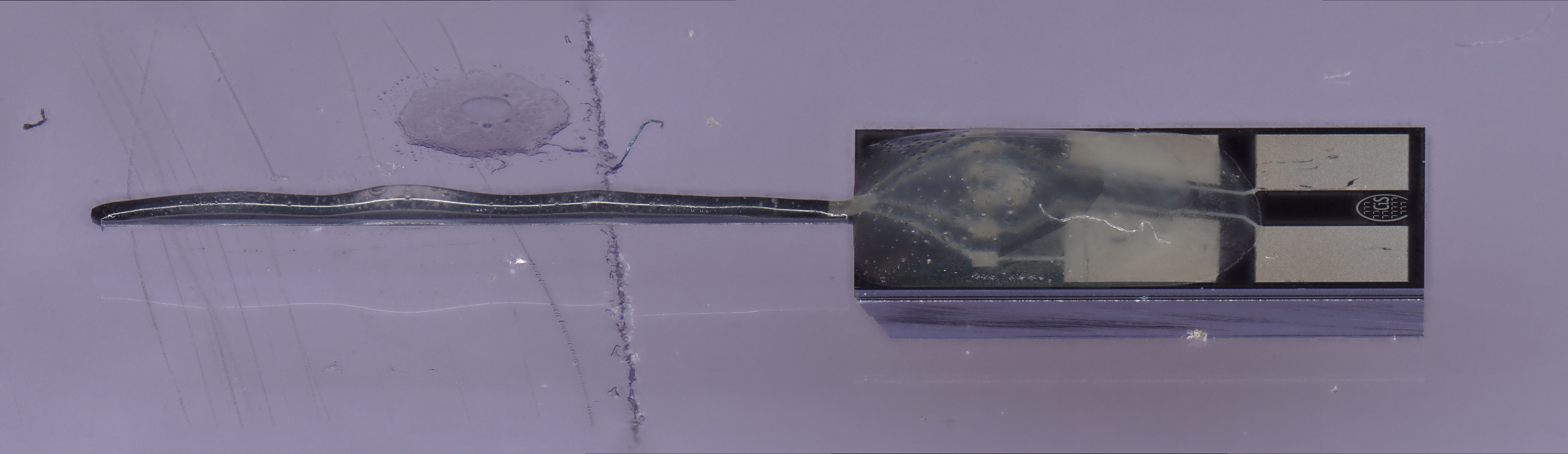

Supplement: Supplementary file 1 [file sensors-23-02003-s001.zip › Figure S15.jpg]

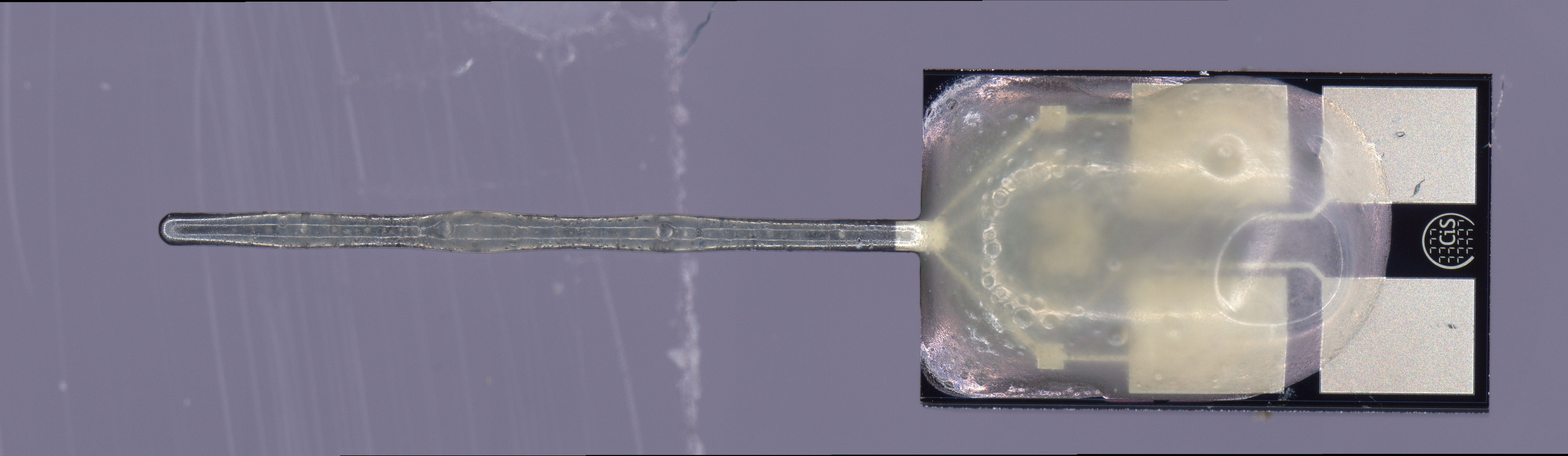

Supplement: Supplementary file 1 [file sensors-23-02003-s001.zip › Figure S16.jpg]

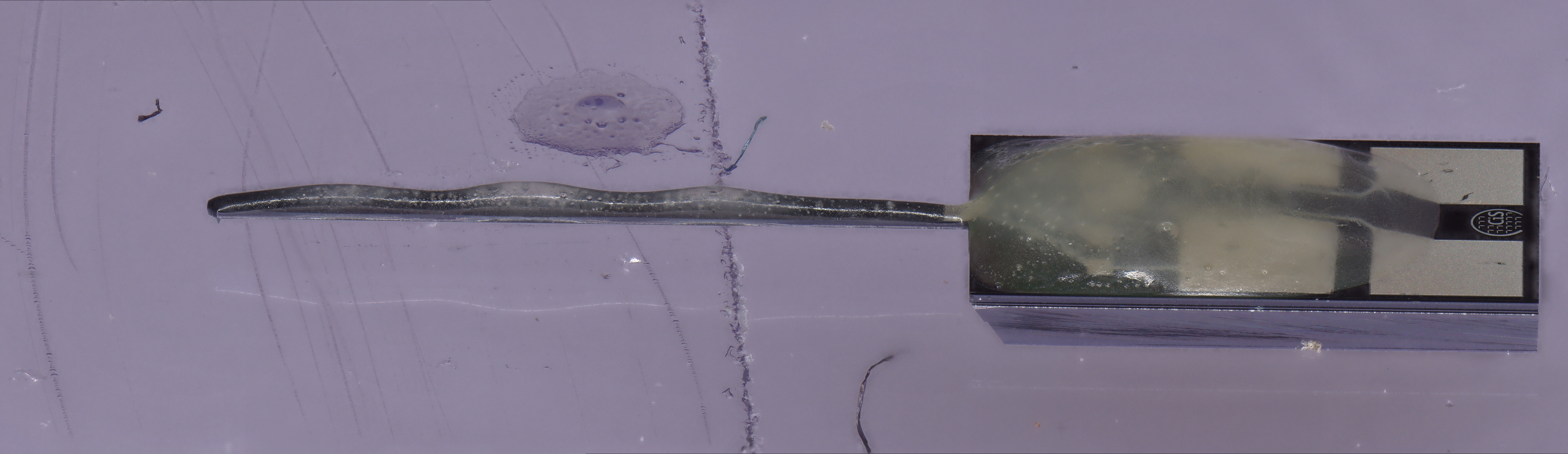

Supplement: Supplementary file 1 [file sensors-23-02003-s001.zip › Figure S17.jpg]

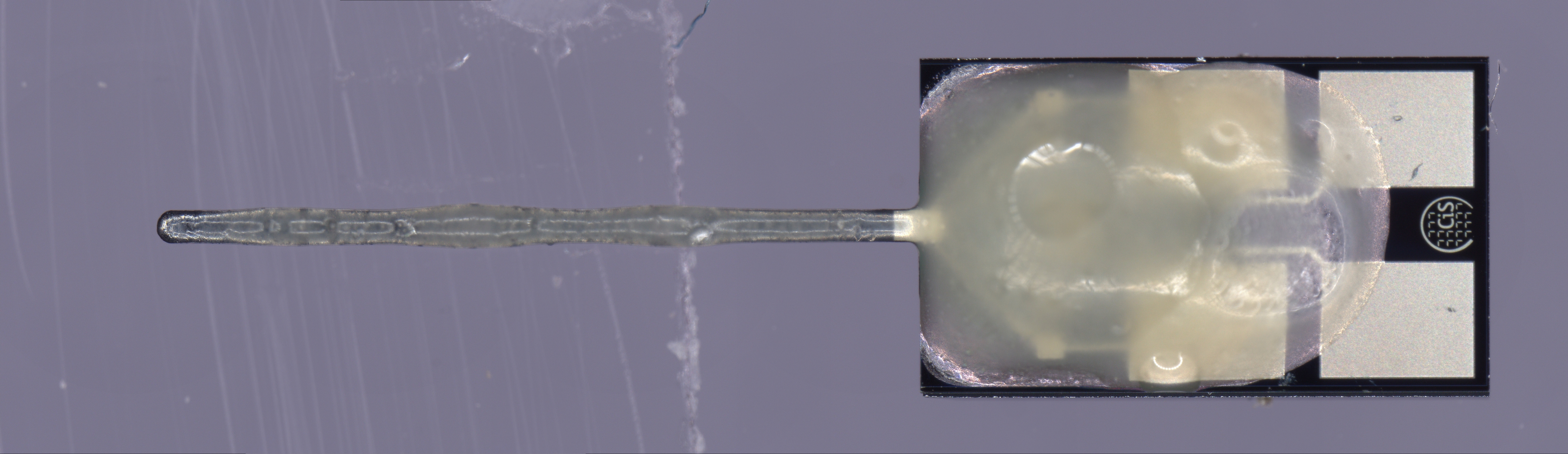

Supplement: Supplementary file 1 [file sensors-23-02003-s001.zip › Figure S18.jpg]

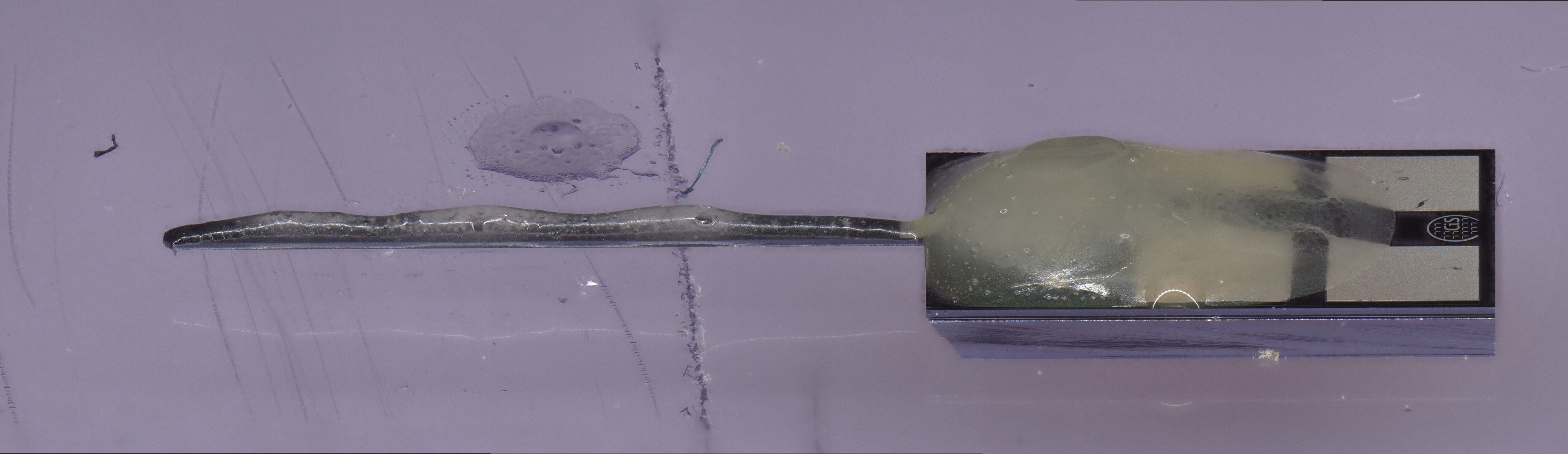

Supplement: Supplementary file 1 [file sensors-23-02003-s001.zip › Figure S19.jpg]

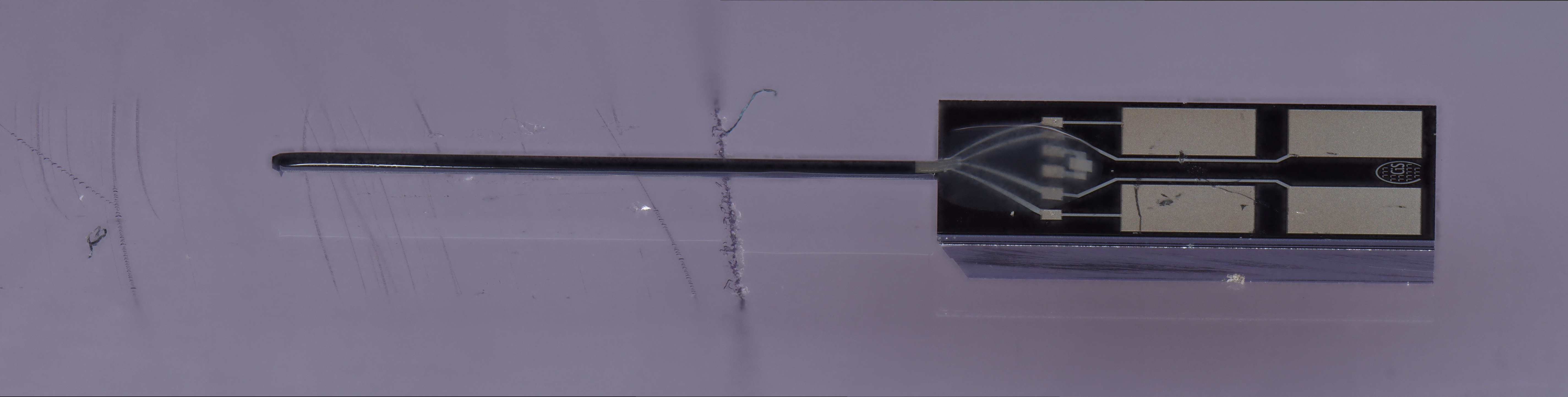

Supplement: Supplementary file 1 [file sensors-23-02003-s001.zip › Figure S2.jpg]

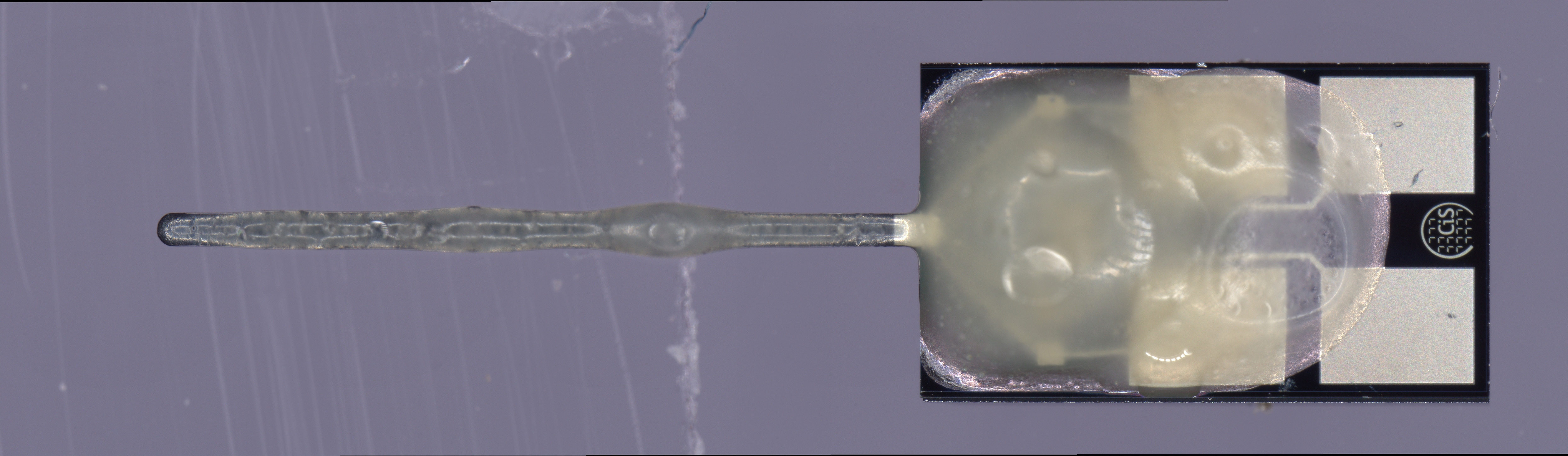

Supplement: Supplementary file 1 [file sensors-23-02003-s001.zip › Figure S20.jpg]

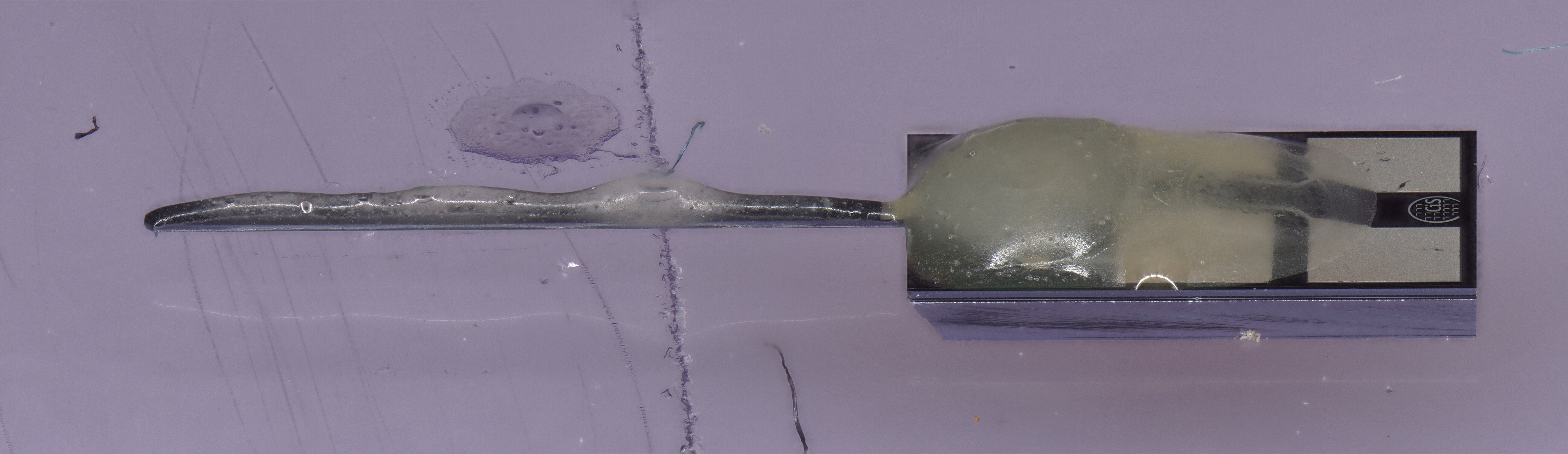

Supplement: Supplementary file 1 [file sensors-23-02003-s001.zip › Figure S21.jpg]

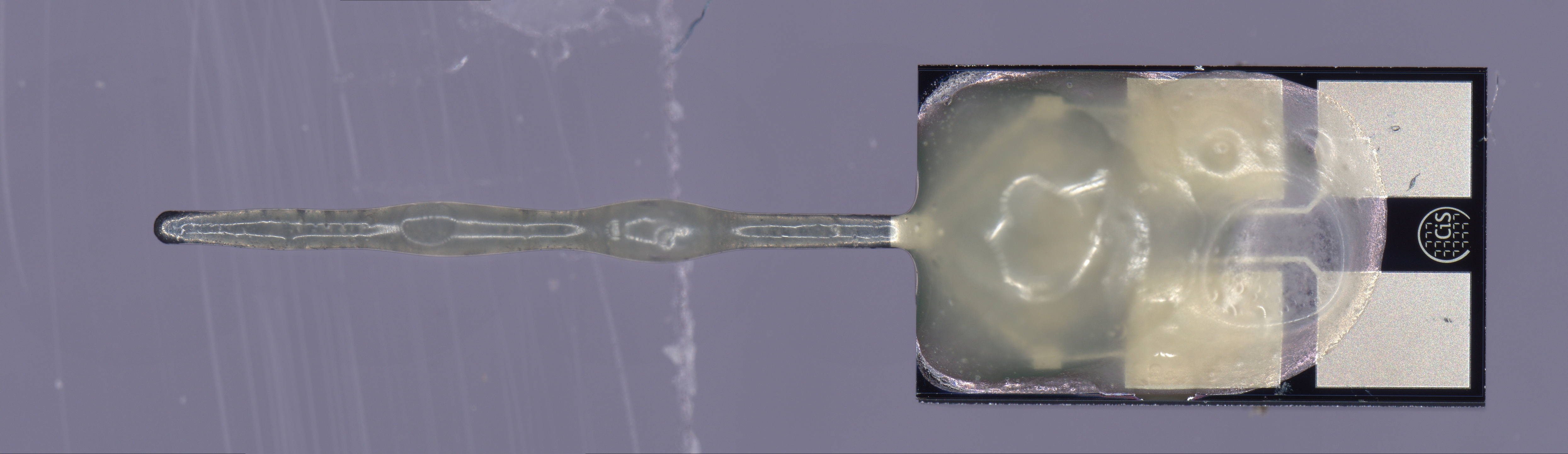

Supplement: Supplementary file 1 [file sensors-23-02003-s001.zip › Figure S22.jpg]

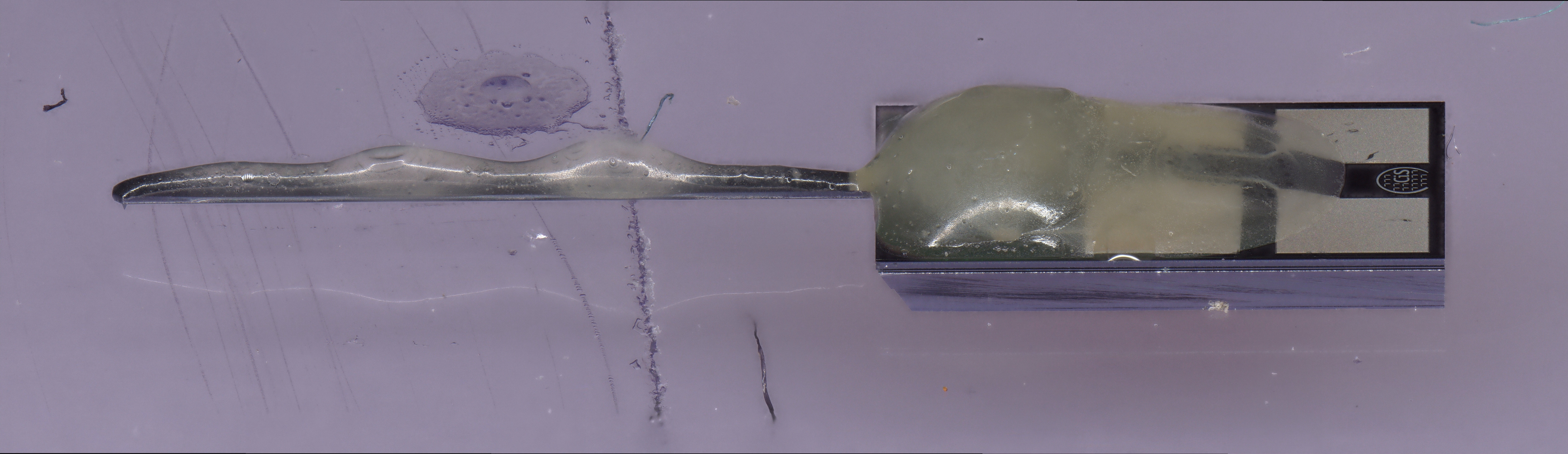

Supplement: Supplementary file 1 [file sensors-23-02003-s001.zip › Figure S23.jpg]

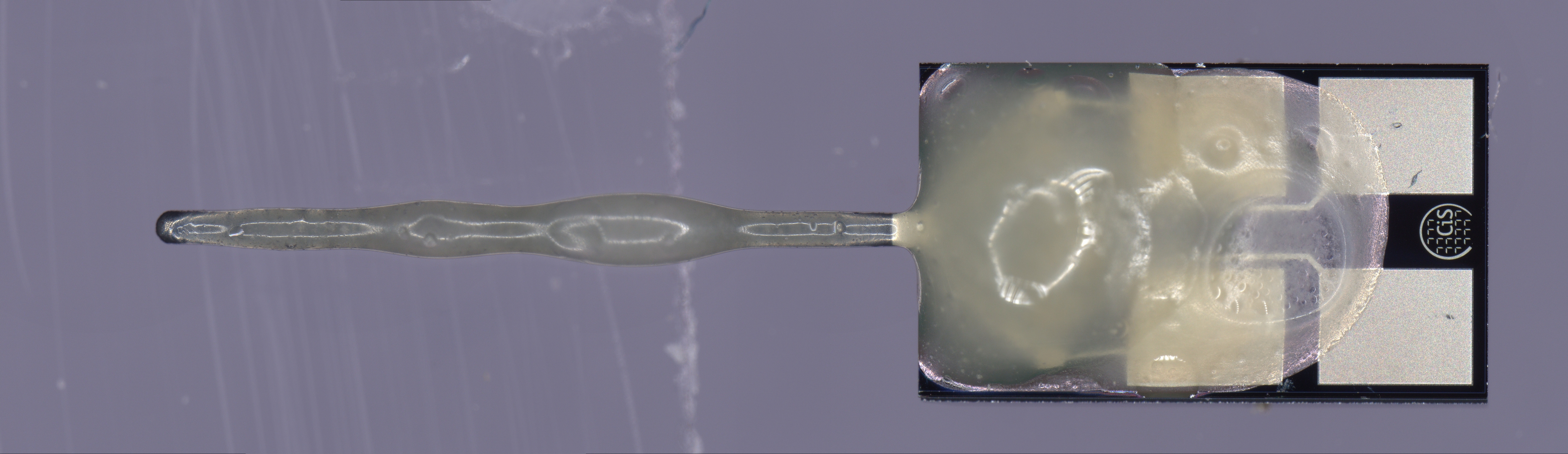

Supplement: Supplementary file 1 [file sensors-23-02003-s001.zip › Figure S24.jpg]

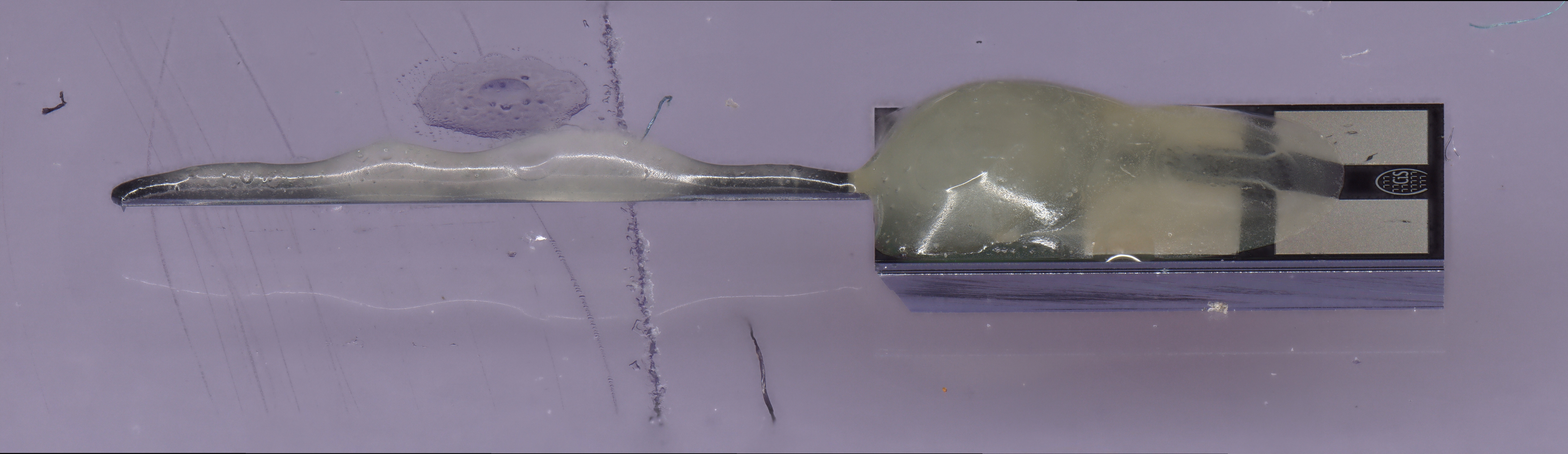

Supplement: Supplementary file 1 [file sensors-23-02003-s001.zip › Figure S25.jpg]

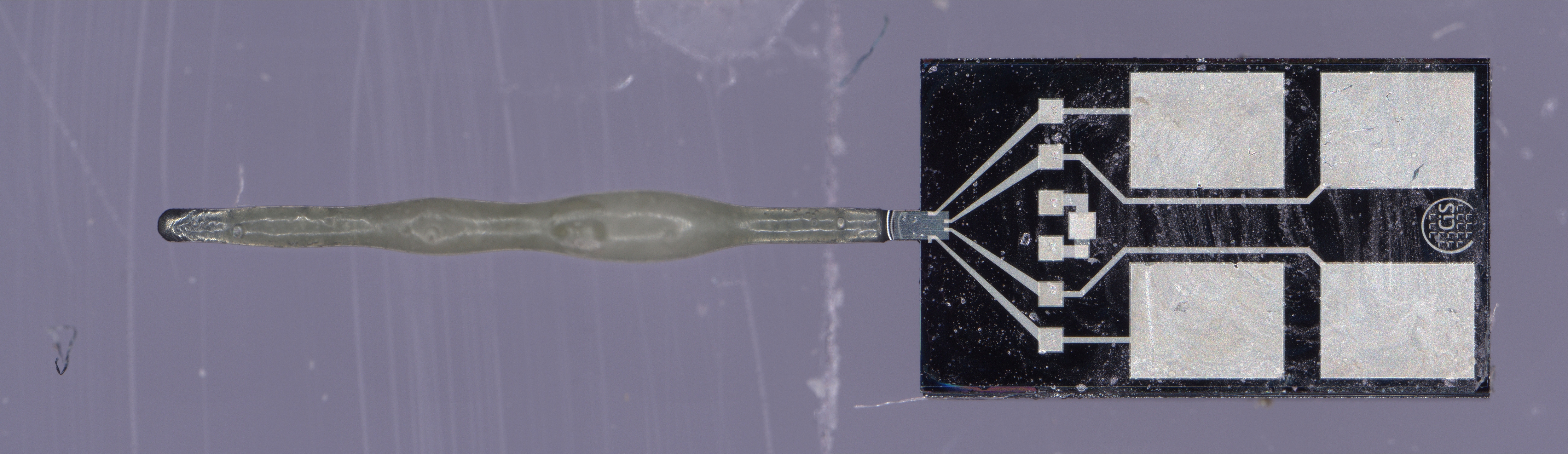

Supplement: Supplementary file 1 [file sensors-23-02003-s001.zip › Figure S26.jpg]

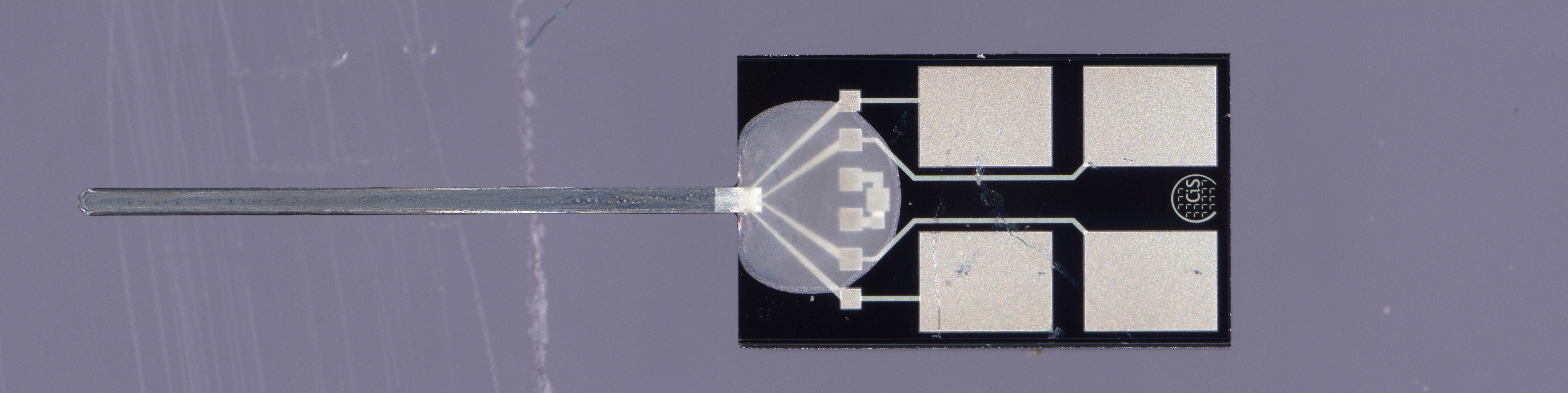

Supplement: Supplementary file 1 [file sensors-23-02003-s001.zip › Figure S3.jpg]

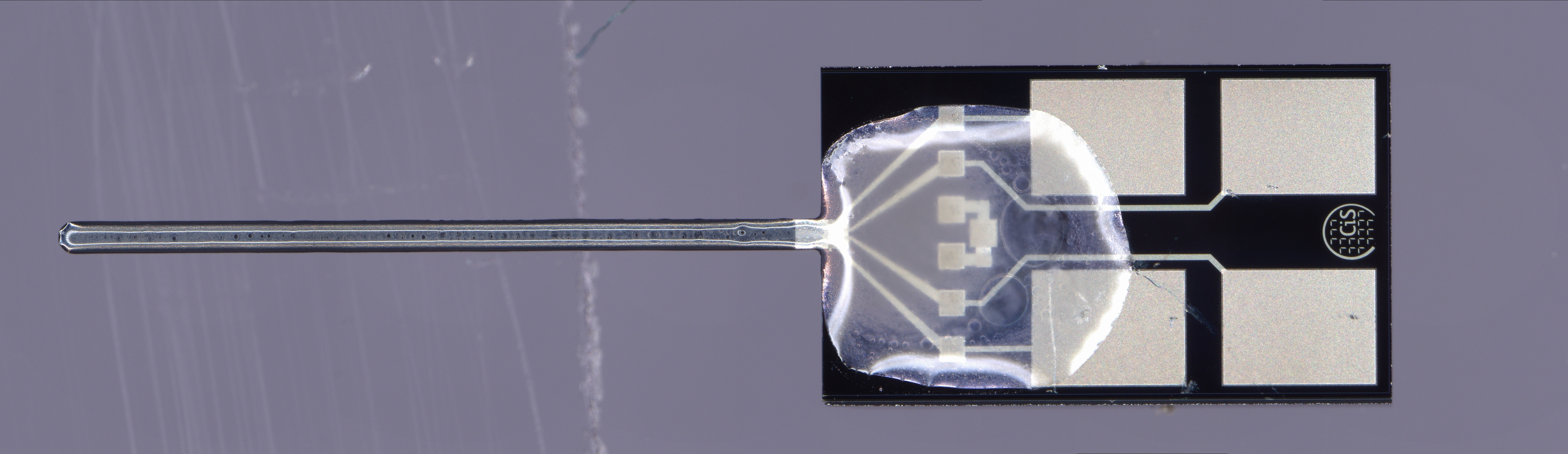

Supplement: Supplementary file 1 [file sensors-23-02003-s001.zip › Figure S4.jpg]

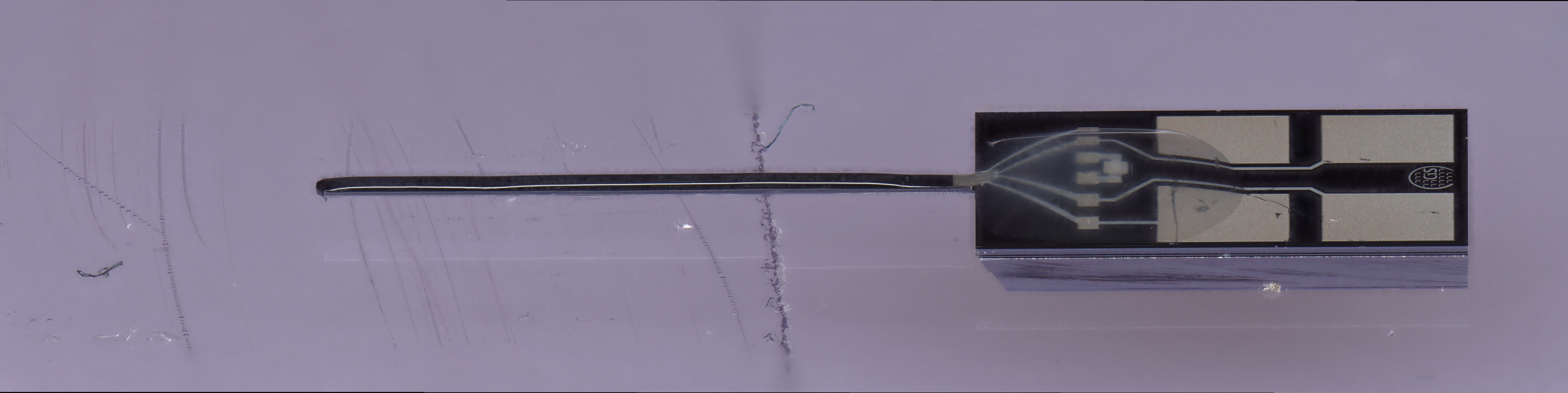

Supplement: Supplementary file 1 [file sensors-23-02003-s001.zip › Figure S5.jpg]

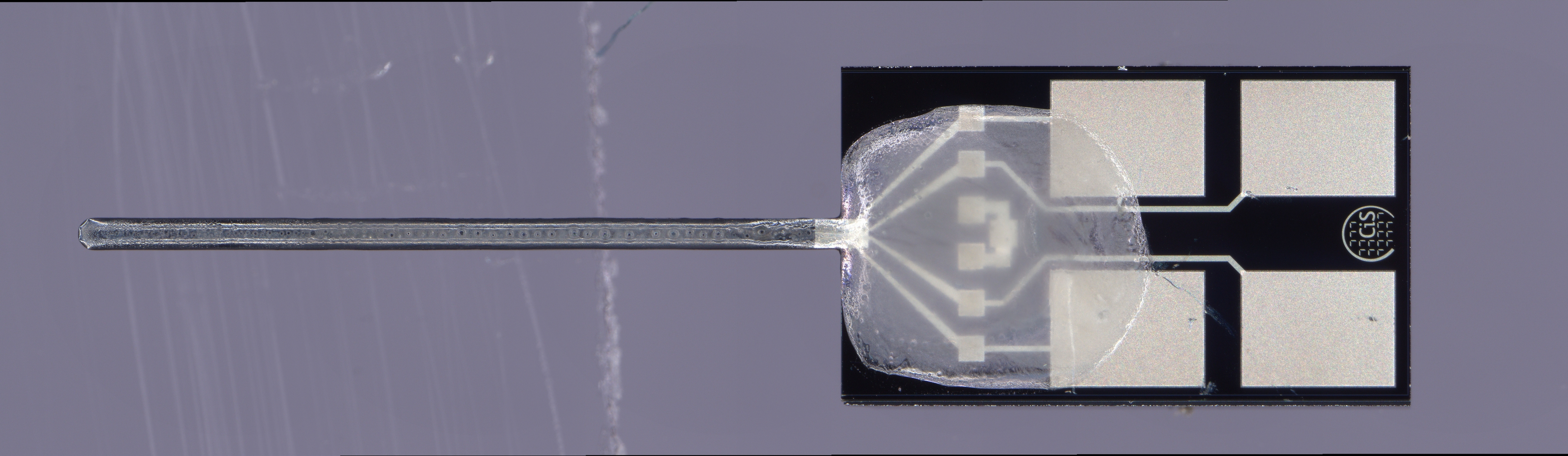

Supplement: Supplementary file 1 [file sensors-23-02003-s001.zip › Figure S6.jpg]

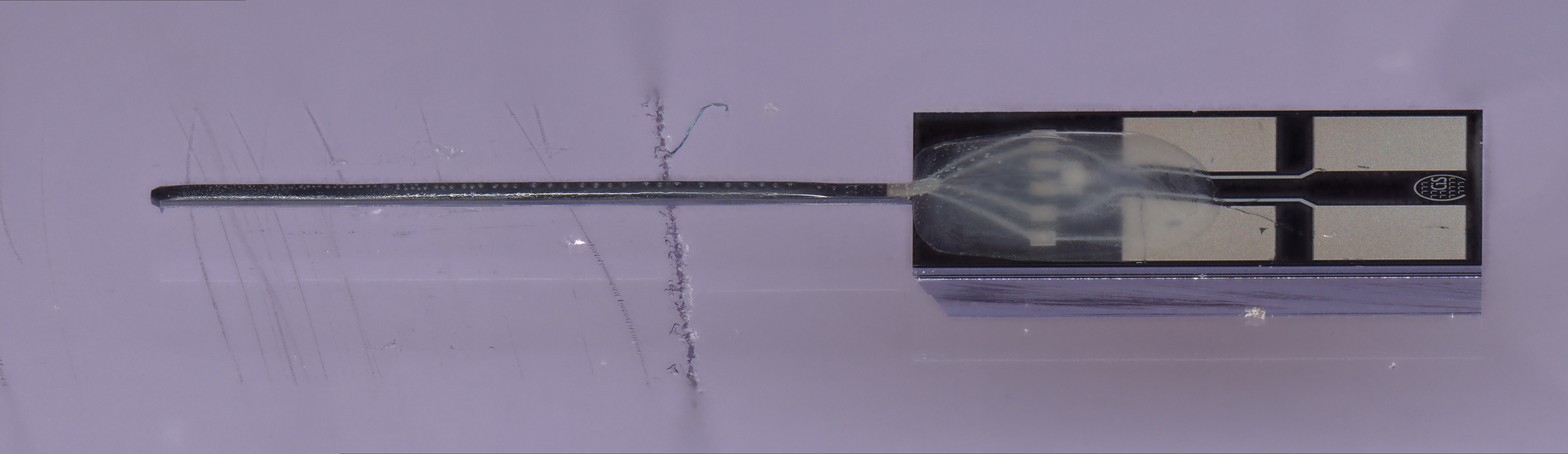

Supplement: Supplementary file 1 [file sensors-23-02003-s001.zip › Figure S7.jpg]

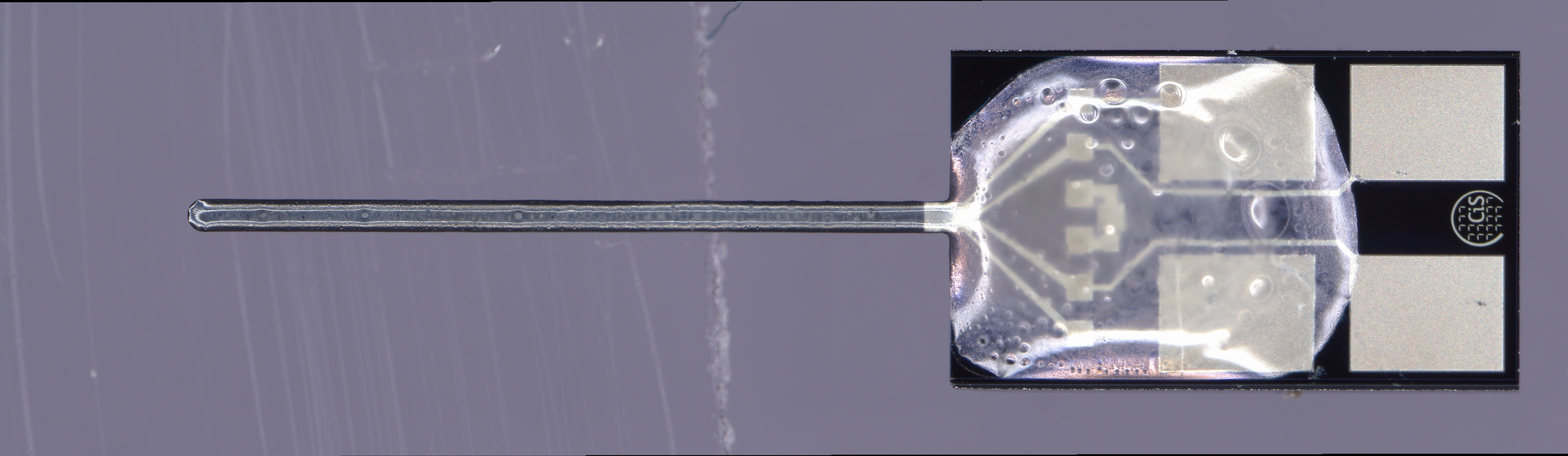

Supplement: Supplementary file 1 [file sensors-23-02003-s001.zip › Figure S8.jpg]

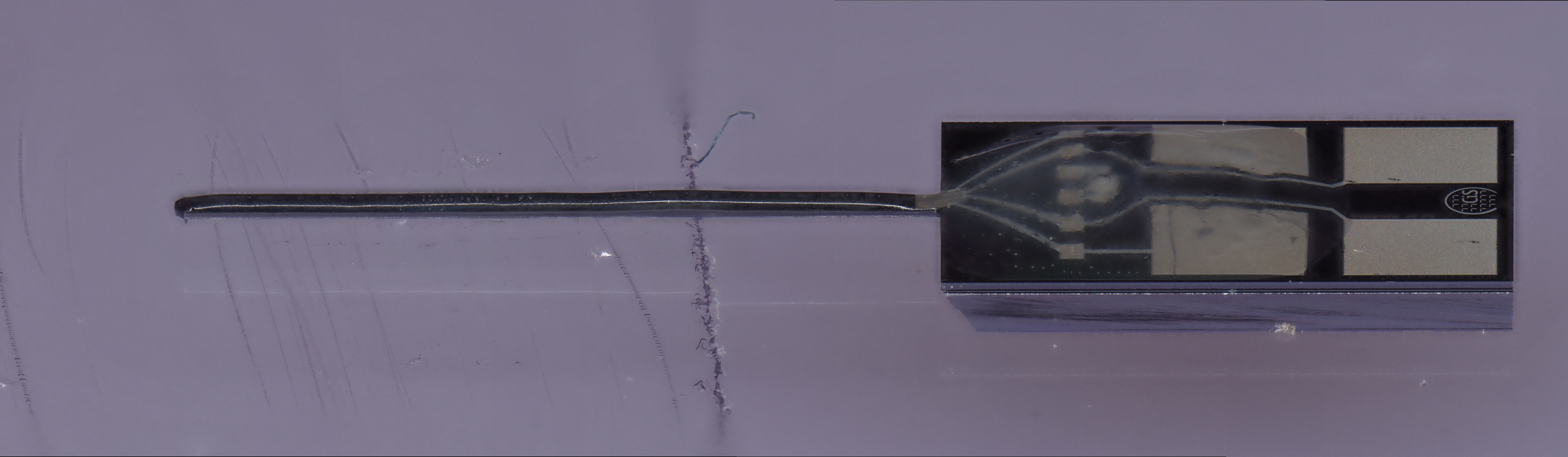

Supplement: Supplementary file 1 [file sensors-23-02003-s001.zip › Figure S9.jpg]
